# Genomic Diversity, Population Structure, and Signature of Selection in Five Chinese Native Sheep Breeds Adapted to Extreme Environments

**DOI:** 10.3390/genes11050494

**Published:** 2020-04-30

**Authors:** Adam Abied, Alnoor Bagadi, Farhad Bordbar, Yabin Pu, Serafino M.A. Augustino, Xianglan Xue, Feng Xing, Gebremedhin Gebreselassie, Jian-Lin Han, Joram M. Mwacharo, Yuehui Ma, Qianjun Zhao

**Affiliations:** 1 Institute of Animal Science (IAS), Chinese Academy of Agricultural Sciences (CAAS), Beijing 100193, China; aa.abied89@gmail.com (A.A.); farhadnevergiveup@yahoo.com (F.B.); puyabin@caas.cn (Y.P.); larrykazunari@126.com (X.X.); gerageruggg@gmail.com (G.G.); yuehui.ma@263.net (Y.M.); 2 Dry Land Research Center (DLRC) and Animal Production, Agricultural Research Corporation (ARC), Wad Madani 511, Sudan; alnoorbagadi@gmail.com; 3 College of Animal Science and Technology, China Agricultural University (CAU), Beijing 100193, China; serafino156@gmail.com; 4 College of Animal Science, Talimu University (TU), Xinjiang, Alar 843300, China; xingfeng2001@126.com; 5 CAAS-ILRI Joint Laboratory on Livestock and Forage Genetic Resources, Institute of Animal Science, Chinese Academy of Agricultural Sciences (CAAS), Beijing 100193, China; h.jianlin@cgiar.org; 6 Livestock Genetics Program, International Livestock Research Institute (ILRI), Nairobi 00100, Kenya; 7 International Center for Agricultural Research in the Dry Areas (ICARDA), Addis Ababa 1108-2010, Ethiopia; j.mwacharo@cgiar.org

**Keywords:** high density SNPs, genomic diversity, selection sweep, Chinese sheep, adaptation

## Abstract

Through long term natural and artificial selection, domestic sheep (*Ovis aries*) have become adapted to a diverse range of agro-ecological environments and display multiple phenotypic traits. Characterization of diversity and selection signature is essential for genetic improvement, understanding of environmental adaptation, as well as utilization and conservation of sheep genetic resources. Here, we aimed to assess genomic diversity, population structure, and genomic selection among five Chinese native sheep breeds using 600K high density SNP genotypes. A total of 96 animals of the five breeds were selected from different geographical locations with extremely dry or humid conditions. We found a high proportion of informative SNPs, ranging from 93.3% in Yabuyi to 95.5% in Wadi, Hu, and Hetian sheep. The average pairwise population differentiation (F_ST_) between the breeds was 0.048%, ranging from 0.022% to 0.054%, indicating their low to moderate differentiation. PCA, ADMIXTURE, and phylogenetic tree analyses revealed a clustering pattern of the five Chinese sheep breeds according to their geographical distribution, tail type, coat color, body size, and breeding history. The genomic regions under putative selection identified by F_ST_ and XP-EHH approaches frequently overlapped across the breeds, and spanned genes associated with adaptation to extremely dry or humid environments, innate and adaptive immune responses, and growth, wool, milk, and reproduction traits. The present study offers novel insight into genomic adaptation to dry and humid climates in sheep among other domestic animals and provides a valuable resource for further investigation. Moreover, it contributes useful information to sustainable utilization and conservation of sheep genetic resources.

## 1. Introduction

Adaptation of livestock breeds to local climatic conditions is an important trait for contemporary agriculture because it reduces environmental stress on animals and leads to an increased and more environmentally friendly production [1]. With around 1000 breeds existing worldwide in various environments, e.g., hot and cold climates, domestic sheep (*Ovis aries*) are an excellent model to study genetic adaptation. Extreme environmental conditions are a major challenge to livestock production. Changes in climatic conditions, particularly those contributing to weather extremes, like drought or extreme temperature or humidity, may compromise immune functions and thus result in poor reproduction and production performance of domestic animals [2]. Different climate zones have a long-term impact on the adaptive evolution of the major sheep genetic lineages in China [3]. Sheep have spread and become adapted to a wide range of agro-ecological environments, especially those distributed on plateaus or in desert or humid regions, because they are vulnerable to climate change [4,5]. However, the influence of different husbandry cultures on the phenotypes of modern sheep breeds is not well understood. In fact, sheep are uniquely domesticated animals which are managed under production systems with extremely different environmental and agro-ecological conditions (e.g., humid versus dry), they could serve as an appropriate evolutionary model, that allows us to gain novel insight into the understanding of genetic basis on how domestic animals have adapted to extreme environments in a short period of time after their domestication, so that to develop appropriate breeding programs under scenarios of future climate changes [6,7].

Natural selection plays an important role in determining the individuals that are best adapted to any environments. Besides natural selection, artificial selection has been widely applied to livestock species/populations to achieve more desirable and profitable traits [8]. Sheep domestication dates back to the end of the Mesolithic period, ~11,000 year ago, making it one of the first domesticated herbivores [9]. Moreover, domestication is the process, following which the protection offered by domestic habitat suppresses the original environmental adaptation. Sheep have played an important role in human society and spread globally [10]. Domestication, natural and artificial selectin have led to marked changes in sheep behavior, appearance, and other important traits [11]. China is rich in sheep resources that have been developed over a long history of sheep breeding [12,13]. Based on the tail type, the Chinese native sheep breeds are divided into five types: Short fat-tailed sheep, long fat-tailed sheep, short thin-tailed sheep, long thin-tailed sheep, and fat-rumped sheep. According to historical, archaeological, and genetic evidence, Mongolian sheep is the common ancestor of Chinese short fat-tailed sheep breeds. Following trading, inter-ethnic wars, and the southward migration of steppe tribes, Mongolian sheep have been introduced into different parts of China, including Gansu, Xinjiang, Qinghai, Henan, Zhejiang, Jiangsu, and Shandong among other provinces. Hu sheep from Jiangsu and Wadi sheep from Shandong belong to Mongolian sheep group [13]. Hetian and Yabuyi sheep are short fat-tailed breeds in Xinjiang while Karakul sheep is an improved breed with a long fat-tail present also in Xinjiang. However, following their distribution from the Mongolian plateau to different agro-ecological regions around almost the entire country, Mongolian sheep have experienced remarkable changes in climatic, environmental, and feeding conditions [13]. All of these factors have the potential to drive the changes in adaptation and selection resulting in microevolution [14,15]. Different breeds belonging to Mongolian sheep group show significant variations in a number of traits, especially related to reproduction, but how these breeds differ genetically in relation to those traits is poorly understood [13]. Genetic diversity represents fundamental basis for adaptation and breeding [16,17]. It is important to document the relative levels of genetic diversity within and between these breeds, so as to provide useful information for breeding and conservation programs.

Detection of signature of selection has potential to reveal new insight into the mechanism of contemporary breeding and artificial selection as well as the causal genes associated with phenotypic variations and important traits. Study on locally adapted breeds is promising to underpin the genes involved in ecologically and economically important traits. Signatures of selection are of great interest in the context of breed differentiation [17]. Several methods for the detection of genomic regions that have undergone selection exist and have recently been applied to a number of wild and domestic sheep populations [12,17,18,19,20,21,22,23,24,25,26,27,28]. Although the genetic basis underlying economically important traits in sheep have been widely studied, our knowledge of the genetic mechanism responsible for adaptation to local environments is still scarce. This study aimed to assess the genomic diversity and population structure, and to detect selection signature in the genomes of the five Chinese local sheep breeds adapted to extremely dry or humid environments using high-density SNP genotyping data. Potential biological functions of the genes with strong selection signals were examined using multi-level bioinformatics approaches.

## 2. Materials and Methods

### 2.1. Ethics Statement

All animal experiments in this study were fully approved by the Animal Care and Use Committee of the Institute of Animals Science, Chinese Academy of Agricultural Sciences (IAS-CAAS) with the following reference number: IASCAAS-AE-03, on 1 September 2014.

### 2.2. Samples Collection and DNA Extraction

A total of 96 Chinese sheep from five diverse indigenous breeds were selected. Ear tissue or whole blood samples were collected from Hetian, Karakul, and Yabuyi sheep distributed in dry environment and Hu and Wadi sheep in humid environment (Table S1). Ear tissue samples were collected into 2-mL micro-centrifuge tubes with 75% ethanol. Genomic DNA was extracted from the ear tissue using standard phenol chloroform method. Around 10 mL of blood was collected from the jugular vein of each animal into vacutainer tubes containing EDTA as anticoagulant and stored in ice/liquid nitrogen (−196 °C). DNA from the whole blood was extracted using DNeasy Blood and Tissue Kit (Qiagen, Dusseldorf, Germany). The concentration and purity of the DNA were measured using NanoDrop 2000 spectrophotometer (Thermo Fisher Scientific Inc., Waltham, MA, USA) and stored at −20 °C for genotyping.

### 2.3. Genotyping and Quality Control

The DNA samples were genotyped using Ovine Infinium HD SNP BeadChip (Beijing KPS Biotechnology Co Ltd., China), which contains 606,006 SNPs. We performed quality control (QC) of these SNPs using PLINK v.1.9 software [29] following the criteria listed below [30]. SNPs or individuals were removed if any of the following criteria were met: (1) No chromosomal or physical location; (2) Minor allele frequency (MAF) < 0.05 within the breeds; (3) SNPs with a call rate < 0.95; (4) Missing genotyping frequency > 0.02; and (5) *p*-value for Hardy-Weinberg equilibrium < 0.00001. After QC filtering, the final dataset consisted of 502,072 autosomal SNPs and 96 animals.

### 2.4. Genomic Diversity and Population Structure Analyses

To understand the population structure within and between the breeds, various parameters related to genomic diversity and population structure, including observed (H_O_) and expected (H_E_) heterozygosity for each breed, were calculated with the command --het as implemented in the PLINK, and inbreeding coefficient (F_IS_) was measured with the command –F also in the PLINK. Genomic relationships represented by the genome-wide proportions of shared identical-by-descent alleles were obtained for each pair of samples using the --genome options in the PLINK. The ADMIXTURE v.1.3. program [31] was employed to determine the population structure clustering based on the optimal number of K clusters. The number of assumed ancestral genetic clusters (K) ranged from 1 to 10, with 10,000 iterations for each run. Apart from the model-based clustering analysis, we further investigated the population structure using principle component analysis (PCA). To examine the genetic relationships, a neighbor-joining (NJ) phylogenetic tree was reconstructed with Reynold’s genetic distances using MEGA7 package [32]. Also, a maximum likelihood (ML) tree was reconstructed using RAxML version 8.2.12 program [33] with 1000 bootstrap replicates and the GTRGAMMA model of nucleotide substitution followed by a correction for ascertainment bias (-m ASC_GTRGAMMA-asc-corr = stamatakis -f a -# 1000). In addition, pairwise F_ST_ values between the breeds were derived using the --fst option in the PLINK. To better understand the population variation, LD decay patterns, which can inform population demography, were investigated for each breed with r2 values calculated with the parameter --r2--ld--window 1000--ld--window--r2 0 command in the PLINK.

### 2.5. Genome-Wide Selection Signatures

We preformed selection signals across the genomes using fixation index (F_ST_) and cross population extended haplotype homozygosity (XP-EHH) approaches. We categorized the sheep breeds into two groups: extremely humid (Hu and Wadi sheep) versus extremely dry (Hetian, Krarakul, and Yabuyi sheep) environments as well as short fat-tailed (Yabuyi sheep) versus long fat-tiled (Karakul sheep) groups. The 100 kb window size with 50 kb sliding step were used to avoid windows with less than five SNPs, which may bias the estimation of the parameters used to detect selection sweeps [34]. To detect selection signals, we calculated the genome-wide distribution of F_ST_ values [35] for each SNP in each window between the groups following previously reported procedures [36]. The F_ST_ values were Z-transformed and the Z(F_ST_) ≥ 5 was considered as the threshold to identify selection signatures [18].

We also compared the extended haplotype homozygosity (EHH) between the groups of Chinese local sheep breeds (humid versus dry environments and short-tailed versus long fat-tailed sheep) using the XP-EHH statistic [37] implemented in the rehh package [38]. The XP-EHH tests can evaluate if a given genomic site is homozygosity in one population but polymorphic in another by comparing the EHH scores at one core SNP of the two populations [39], without considering ancestral information. XP-EHH of a given focal SNP was defined and standardized according to Sabeti et al. [37] and Gautier et al. [40] and transformed to PXP-EHH following Gautier and Naves [41]. As PXP-EHH may be interpreted as a two-sided *p*-value in a –log10 scale, candidate genomic regions with a *p*-value ≤ 0.01 (1%) were considered as signals of selection in the test. A negative XP-EHH score suggests selection in the reference population while a positive score indicates selection in the observed population. XP-EHH is believed to have a high power in detecting selection signatures with almost or fully fixed haplotypes and also approximately follow a standard normal distribution [37].

### 2.6. Annotation of Highly Significant Genomic Regions

To improve the confidence in the selected outlier windows of putative genomic regions under selection, the top 1% SNPs showing high statistical significances and which overlapped between F_ST_ and XP-EHH analyses were considered. The genomic locations of such SNPs were extended up to 50 kb upstream and downstream of each of the most significant SNPs to search for overlapping genes based on the version Oar4.0 sheep reference genome assembly (https://www.ncbi.nlm.nih.gov/assembly/GCA_000298735.2). These genes and their associated annotations were downloaded from the annotated sheep genome in the Ensemble database (https://www.ensemble.org) and used for functional enrichment analysis.

### 2.7. Functional Enrichment Analysis of Candidate Genes

The DAVID platform [42,43] and a web-based toolset: g: profiler (https://biit.cs.ut.ee/gprofiler/sheep) were used to perform the functional enrichment analysis of the candidate genes under selection. Further investigation of the biological functions of the candidate genes was inferred through a search of literature using the NCBI database (https://www.ncbi.nlm.nih.gov/gene/) to describe the most over-represented pathways and biological processes affected by the candidate genes.

## 3. Results

### 3.1. Genomic Diversity Analysis

**Figure 1:**
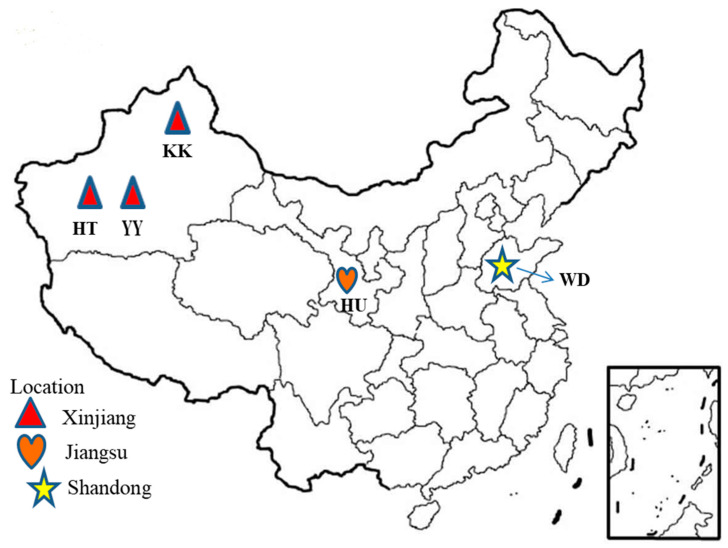
Map of China showing the sampling locations of the five Chinese local sheep breeds analyzed in this study.

In total, 96 individuals from the five Chinese local sheep breeds sampled in different geographical environments (humid versus dry) (Figure 1) and with unique phenotypic characteristics (short fat-tailed versus long fat-tailed) were genotyped using the 660K Ovine Infinium HD SNP BeadChip. After the QC filtering, the final numbers of animals and SNPs retained for all downstream analyses were 96 and 502,072, respectively. Genomic diversity within each of the five breeds were assessed by estimating the percentage of polymorphic SNPs, H_O_, H_E_, minor allele frequency (MAF), and F_IS_ (Table 1). The percentages of polymorphic SNPs ranged from 93.5% in Yabuyi to 95.5% in Hetian sheep. The average H_O_ was the lowest in both Yabuyi and Wadi (0.360) but the highest in Hetian (0.367) sheep. The H**_E_** ranged from 0.356 in Yabuyi to 0.360 in both Wadi and Hetian sheep. The F_IS_ varied between −0.03 in Karakul and −0.004 in Wadi sheep. The fixation index (F_ST_) was used to measure of the homogeneity of samples between each breed pair, where the higher value indicates a greater differentiation between the two breeds due to genetic structure. F_ST_ ranged from 0.022 between Hetian and Yabuyi sheep to 0.054 between Karakul and Yabuyi (Table S2) sheep.

### 3.2. Population Relationship and Structure Analyses

**Figure 2:**
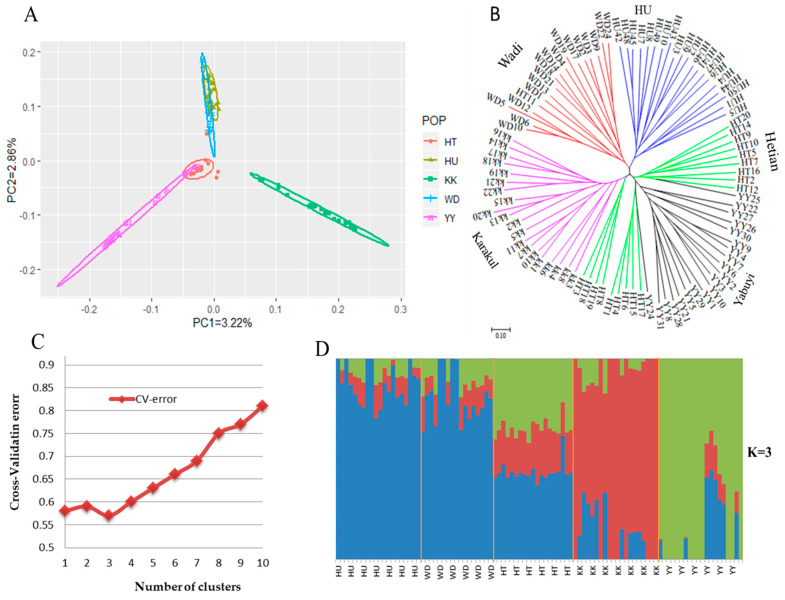
Population relationship and structure analyses of the five Chinese local sheep breeds from extremely dry (HT, YY, and KK) and humid (WD and HU sheep) environments. (**A**) Principal component (PC) analysis among the 96 sheep of the five breeds. (**B**) Maximum-likelihood tree reconstructed using the RAxML with the GTRGAMMA model followed by a correction for ascertainment bias. (**C**) Cross-validation errors across the 10 assumed ancestral genetic clusters among the five breeds. (**D**) Genome-wide admixture proportions at K = 3 among the 96 sheep of the five breeds.

To assess the genetic relationship and structure among the five Chinese local sheep breeds, PCA (Figure 2A), ML tree (Figure 2B and Figure S3), NJ tree (Figure S4), and ADMIXTURE (Figure 2D) analyses were performed. The first two principal components, explaining 6.08% of the total variation, were used to visualize the relationship among the five Chinese sheep breeds from humid and dry environments. The result showed that the breeds from the humid environment (Hu and Wadi sheep) clustered closely together and they were clearly separated from the breeds from the dry environment (Hetian, Karakul, and Yabuyi sheep). Furthermore, the breeds from the dry environment were separated into Hetian and Yabuyi sheep in one group and Karakul sheep in another group, which were in accordance to their coat colors, tailed-types, geographical distribution, and domestication history (Figure 2A). The ML (Figure 2) and NJ (Figure S4) trees indicated that Hu, Wadi, and Karakul sheep were genetically distinct breeds, whereas Hetian sheep seemed to be subdivided into three clusters, of which one cluster aligned with Karakul sheep while the other two clusters mixed with Yabuyi sheep (Figure 2B). However, in the ML-bootstrapping tree (Figure S3), some of these clusters had low values of bootstraps and their subdivisions were thus not fully statistically supported.

To further analyze the population structure, we investigated population admixture using the ADMIXTURE with K values ranging from 1 to 10 (Figure 2C and Figure S1). Based on the cross-validation errors, K = 3 was identified to be the most optimal number of genetic clusters defining the population structure among the five Chinese local sheep breeds (Figure 2C,D). One genomic cluster was predominant in Hu and Wadi sheep, and the other two clusters were observed in Karakul and Yabuyi sheep. Hetian sheep was the most admixed and showed all the three genetic clusters with none predominating. At K = 4 and 5, such genetic admixture was observed among all the five sheep breeds (Figure S1).

### 3.3. Detection of Selection Signatures

In this study, we calculated the genome-wide distribution of the F_ST_ and XP-EHH values, to explore the selection signatures between the groups of breeds from extreme ecological environments and with different tail types.

**Figure 3:**
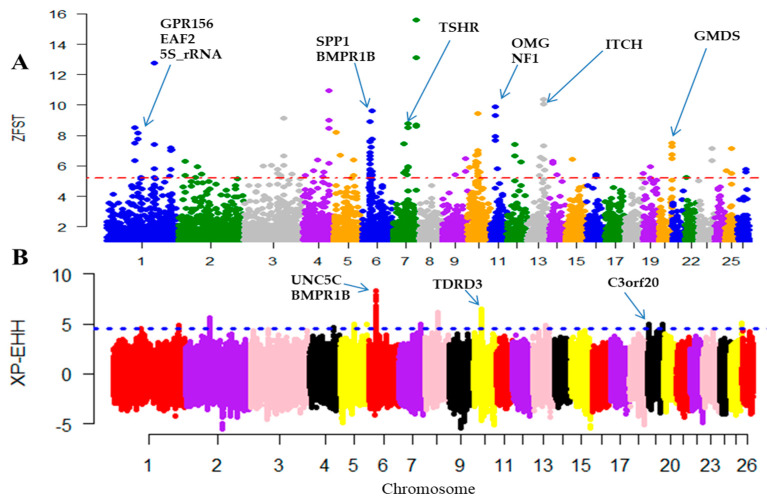
Manhattan plots of selection signatures determined by comparing the five Chinese local sheep breeds from the dry and humid environments using the Z(F_ST_) (**A**) and XP-EHH (**B**) approaches.

Gene ontology (GO) enrichment analysis was performed for the candidate genes revealed in pairwise comparisons between the breeds from extreme (humid versus dry) environments. Several genomic regions with high F_ST_ values were detected (Figure 3A). The average F_ST_ across the genomes was 0.0377, indicating little to moderate genetic differentiation according to Wright’s classification. In total, 331 outlier windows and 17 genomic regions under selection were detected across the 26 autosomes based on the top 1% of transformed Z(F_ST_) values. The strongest candidate regions were located on chromosomes (OAR)7, OAR6, OAR10, OAR1, OAR4, OAR11, and OAR13, which spanned multiples genes, for instance, *TSHR, NAP1L5, RBM26, TSPAN1, VPS50, PEMT,* and *PCED1A* (Table 2 and Table S3). The top three most significant GO terms were associated with endoplasmic reticulum (GO: 0005783; *p*-value = 0.0012), regulation of cell growth (GO: 0001558; *p*-value = 0.019), and positive regulation of extrinsic apoptotic (GO: 1902043; *p*-value = 0.020) terms (Table S4).

We also performed the XP-EHH analysis to reveal genomic regions under selection. In total, 16 candidate regions defined by 351 highest statistically significant SNPs (*p* ≤ 0.05) across the 26 autosomes were identified (Figure 3B). Using the criterion of the top 1% XP-EHH values, the strongest candidate genomic regions were mapped on OAR6, OAR10, OAR8, and OAR2 and spanned multiple genes, such as *RASGEF1B, STK24, AGPAT4,* and *EPB41L4B* gene (Table 3 and Table S5). The top three most significant GO terms were associated with post embryonic development (GO: 0009791; *p*-value = 0.0016), extracellular matrix structural constituent (GO: 0005201; *p*-value = 0.0021), and ECM-receptor interaction (oas04611; *p*-value = 0.0026) (Table S6). Multiple genomic regions with strong selection signatures overlapped between the F_ST_ and XP-EHH approaches, for instance, OAR2, OAR6, OAR10, and OAR7 had common genomic regions with several important genes.

**Figure 4:**
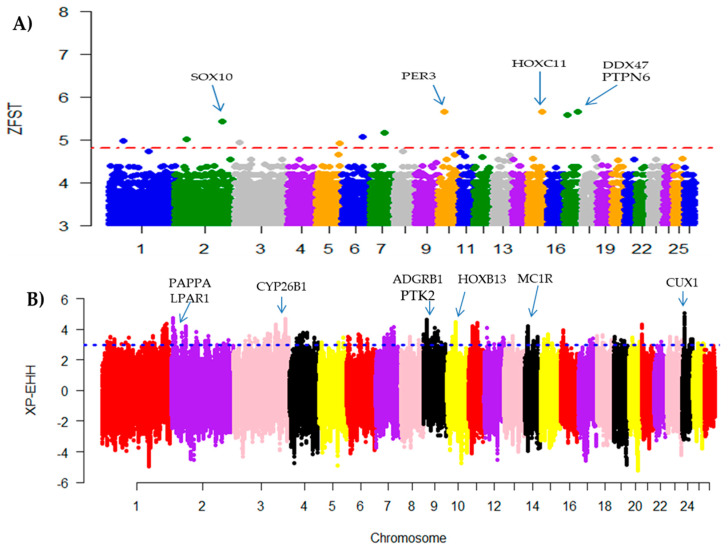
Manhattan plots of genome-wide autosomal Z(F_ST)_ (**A**) and XP-EHH (**B**) analyses for the comparison between the short tailed (Yabuyi sheep) and long fat-tailed (Karakul sheep) breeds.

For the comparison between the short fat-tailed (Yabuyi sheep) and long fat-tailed (Karakul sheep) breeds, multiple genomics regions with high F_ST_ values were observed (Figure 4A). In total, 14 candidate regions were defined by 155 high significant SNPs across the 26 autosomes. Considering the top 1% F_ST_ values, four candidate genomic regions that spanned 25 genes were mapped on OAR2, OAR10, OAR15, and OAR17 (Table 4, Figure 4A). Using the XP-EEH approach, 34 genes embedded in eight candidate genomic regions on OAR24, OAR2, OAR3, OAR9, OAR10, OAR21, and OAR14 were identified from 298 outlier windows across the 26 autosome (Table 5, Figure 4B). The top three most significant GO terms based on the F_ST_ approach included nucleoplasm (GO:0005654; *p*-value = 0.011), extracellular exosome (GO:0070062; *p*-value = 0.014), and anterior/posterior pattern specification (GO:0070062; *p*-value = 0.017) (Table S7). The top three most significant GO terms based on the XP-EHH approach were related to endocytic vesicle (GO:0030139; *p*-value = 0.023), neuronal cell body (GO:0043025; *p*-value = 0.031), and focal adhesion (GO:0005925; *p*-value = 0.031) terms (Table S8).

## 4. Discussion

In the present study, population genomic diversity and genome-wise selection signature analyses were conducted using the 600K SNP genotypes of 96 animals from the five Chinese local sheep breeds adapted to extremely humid and dry environments. We evaluated their population structure using the PCA, ADMIXTURE, and ML and NJ phylogenetic trees. In addition, genome-wide scan of selection signals were analyzed using the F_ST_ and XP-EHH approaches. The results indicated low to moderate genetic variation among the studied sheep breeds. The five Chinese local sheep breeds were divided into three genetic clusters according to their coat colors, body sizes, tail types, geographical locations, and breeding history. Furthermore, multiple genomic regions carrying important candidate genes for adaptation to dry or humid environments were detected.

### 4.1. Genomic Diversity within Breeds

Assessing the within-breed/population genetic variability could provide insight for designing breeding improvement strategies for local sheep genetic resources [12,44]. Hetian, Wadi, and Hu sheep displayed the highest polymorphisms, followed by Karakul and Yabuyi sheep (Table 1). Furthermore, the lowest pairwise differentiation was observed between Yabuyi and Hetain sheep but the highest differentiation was between Yabuyi and Karakul sheep (Table S2). Generally, the levels of genetic polymorphisms in sheep from the humid environment were higher compared to sheep from the dry environment. The H_O_ and H_E_ in Wadi sheep were the same, which might be related to random mating in this breed [45]. In Hetian, Hu, Karakul, and Yabuyi breeds, the H_O_ were higher H_E_ because of regular exchange of breeding rams among flocks within the breeds [45,46]. The lowest genetic diversity was observed in Yabuyi sheep, which could be due to inbreeding following its relatively small population size in an isolated geographical location. Genetic diversity of these five Chinese sheep breeds were higher compared to those observed in Ethiopian fat-tailed sheep [25] and South African indigenous sheep [47], but lower than that of Morada Nova hair sheep breeds in Brazil [48].

### 4.2. Population Structure of Chinese Local Sheep Breeds from Dry and Humid Environments

The PCA, ML and NJ phylogenetic trees, and ADMIXTURE analyses all revealed a common clustering pattern that showed a clear separation between the breeds from humid and dry environments. This pattern may be related to their grazing management and breeding history. Furthermore, the PCA analysis revealed the 96 individuals clustering into three groups on the bases of genetic relationships following their tail types, body sizes and coat colors [12]. Additionally, we found that MAF in different sheep breeds distributed in dry and humid environments were very similar. This result was supported by the low to moderate pairwise F_ST_ differentiations between these breeds. Moreover, breeds form the dry environment formed two separated groups, corresponding to their tail shapes, coat colors, body sizes, and breeding history (Table S1) [12,49,50]. Wadi, Karakul, and Yabuyi sheep displayed the highest LD decay, while Hetian and Hu sheep showed the lowest LD across all genomic distance intervals (Figure S2). In general, the mean r2 values decreased rapidly with increasing genomic distances and attained constantly after 200–300 Kb. The most rapid decrease in r2 was observed in the first five bins, the phenomenon could be due to admixture effects, which was similar with the result previously reported by Gibbs et al. [51].

### 4.3. Selection Signatures of Candidate Genes

Using the F_ST_ and XP-EHH approaches, we identified various genomic regions that are potentially under selection in at least one of the five sheep breeds. It is well known that the detection of common selection signatures by more than one methodology can provide stronger evidence of selection in particular genomic regions [52,53].

#### 4.3.1. Candidate Genes Related to Immune Response and Disease Resistance

Several overlapping genes involved in regulating innate and adaptive immunity in mammals were identified; for example, *HERC2* and *CYFIP1* genes that are relevant to regulate innate and acquired immune responses, as well as cytokine signaling [54]. Multiples candidate genes were also found to participate in host defense mechanism, resistance diseases, and inflammatory responses, with two most significant genes worth to mention as examples. *C5orf30* gene was reported in association with the development of autoimmune disorders in primary biliary cirrhosis as well as the susceptibility and severity of rheumatoid arthritis in humans [55,56]. Another interesting gene is *ITCH,* which encodes a member of the Nedd4 family of *HECT* domain E3 ubiquitin ligases and plays a role in protein trafficking and immune response, and in several signaling pathways that regulate cellular growth and proliferation in multiple processes [57]. A bi-allelic mutation in *ITCH* gene was observed to cause a severe syndromic multisystem autoimmune disease [58].

#### 4.3.2. Candidate Genes Associated with Body Weight and Digestive Metabolism Traits

Growth and body weight are the most economically important traits in livestock breeds that are specialized for meat production. Several breeds from dry or tropical environments tend to have small body weight/size and slow growth rate compared to humid or temperate breeds [59]. Body weight is one of important traits for meat type animals that can be measured at birth or other life stages. Hence, natural and artificial selection may have left traces in the genomic regions harboring genes involved in the production traits of the Chinese sheep from humid and dry environments. We found overlapped candidate genes mapped on OAR6, OAR11, and OAR10. For example, *GJB2* and *GJA3* genes were found to be related to body size and development [19]. *PKD2* gene has been associated with milk production traits [12] and *OMG* gene was involved in metabolism following food deprivation [60]. Centrosomal P4.1-associated protein (*CPAP*) was shown to be involved in the assembly of centrioles and plays a structural role in the maintenance of centrosome function, such as centrosome integrity and normal spindle morphology during cell division, motility, or intracellular traffic [61].

#### 4.3.3. Candidate Genes Associated to Reproduction Traits

Reproduction is a critically important trait in livestock breeding [62] and in the sheep industry particularly [63,64]. It appears to be controlled by multiple genes and factors [65,66], impacting on pathways of ovarian follicular development, embryogenesis, oocyte maturation, ovulation, fertilization, and uterine receptivity.

Interestingly, the most statistically significant gene identified by the comparison of sheep between extreme (dry versus humid) environments was *BMPR1B*, which plays a major role in sheep reproduction and appears in a candidate region on OAR6, that was identified by both F_ST_ and XP-EHH approaches. *BMPR1B* gene is already best-documented for its significant association with prolificacy in sheep [67,68,69] and directly determines litter size in other mammals [70]. *TSHR* gene was identified to be involved in reproduction [20] and *NF1* gene to be associated with litter size [71]. Finally, *BMP2* gene was suggested to be involved in sheep prolificacy and fecundity [72,73].

## 5. Conclusions

In this study, our results revealed low to moderate levels of genetic variability among the five Chinese local sheep breeds distributed in extremely dry and humid environments. Moreover, the five breeds could be divided into three genetic clusters, including breeds from humid environment being one cluster and another two clusters of breeds from the dry environment, following primarily their tail types and geographical distributions. Selection signature analysis identified various candidate genomic regions spanning genes related to skeletal structure and morphology, body temperature regulation, disease resistance, reproduction, and possibly adaptation to local environments. Our findings will provide valuable information to support genomic improvement of these local sheep breeds towards better adaptation to and an increased performance in the extremely dry and humid environments.

## Figures and Tables

**Table 1:** Summary of genotyped animals and genomic diversity estimates in the five Chinese local sheep breeds.

Breed	Code	Location	Sample Size	Ecology	Purpose	P_N (%)_	H_O_	H_E_	F_IS_	MAF
Hetian	HT	Xinjiang	19	Arid land	Meat/Wool	95.5	0.367	0.360	−0.02	0.269
Karakul	KK	Xinjiang	20	Arid/Desert	Lamb skin	94	0.362	0.357	−0.03	0.267
Yabuyi	YY	Xinjiang	20	Arid	Meat	93.5	0.360	0.356	−0.023	0.266
Wadi	WD	Shandong	17	Sub-humid	Meat/Wool	95.3	0.360	0.360	−0.004	0.270
Hu	HU	Jiangsu	20	Humid	Meat/Lamb skin	95.2	0.364	0.358	−0.029	0.268

P_N_: Proportion of polymorphic SNPs; H_O_: Observed heterozygosity; H_E_: Expected heterozygosity; F_IS_: Inbreeding coefficient; MAF: Minor allele frequency.

**Table 2:** Candidate genes in the genomic regions of the top 10 highest peak SNPs based on the F_ST_ approach.

OAR	Gene Position (bp)	Gene Name	Function	Gene Description
1	18,323,4435–18,331,6995	*GPR156*	Defenses	G protein-coupled receptor 156
1	18,456,7855–18,461,9155	*EAF2*	Disease resistance	ELL associated factor 2
1	18,334,3776–18,334,3825	*5S_rRNA*	Disease resistance	5S ribosomal RNA
6	36,566,367–36,630,153	*PKD2*	Milk production	Polycystin 2, transient receptor potential cation channel
6	36,651,734–36,658,288	*SPP1*	Growth/defense/litter size	Secreted phosphoprotein 1
6	29,361,947–29,448,079	*BMPR1B*	Litter size/fecundity/prolifically	Bone morphogenetic protein receptor type 1B
7	89,258,424–89,431,877	*TSHR*	Reproduction	Thyroid stimulating hormone receptor
11	18,317,151–18,318,473	*OMG*	Digestive metabolism	Oligodendrocyte myelin glycoprotein
11	18,245,395–18,441,418	*NF1*	Litter size	Eurofibromin 1
13	63,388,680–63,446,846	*ITCH*	Disease resistance	Itchy E3 ubiquitin protein ligase

**Table 3:** Candidate genes in the genomic regions of the top 10 highest peak SNPs based on the XP-EHH approach.

OAR	Gene Position (bp)	Gene Name	Function	Gene Description
5	49,797,770–49,800,184	*PCDHGA2*	Immunity	Protocadherin gamma subfamily A, 2
5	99,509,492–99,557,151	*C5orf30*	Disease resistance	Chromosome 5 open reading frame 30
6	29,146,028–29,348,864	*UNC5C*	Reproduction	Unc-5 netrin receptor C
6	29,361,947–29,448,079	*BMPR1B*	Litter size	Bone morphogenetic protein receptor type 1B
10	75,064,027–75,351,616	*DOCK9*	Diseases resistant	Dedicator of cytokinesis 9
10	18,352,91–20,655,11	*TDRD3*	Metabolism	Tudor domain containing 3
10	29,454,677–29,502,617	*RXFP2*	Hair/wool	Relaxin family peptide receptor 2
13	48,462,232–48,472,599	*BMP2*	Fecundity	Bone morphogenetic protein 2
19	57,395,864–57,446,138	*C3orf20*	Adaptive immune	Chromosome 3 open reading frame 20
25	43,127,841–43,132,260	*C10orf71*	Adaptive immune	Chromosome 10 open reading frame 71

**Table 4:** The candidate genomic regions and genes identified to be under selection by the F_ST_ approach for the comparison between the short fat-tailed (Yabuyi sheep) and long fat-tailed (Karakul sheep) breeds.

OAR	Gene Position (bp)	GENE NAME	Function
10	35,583,842–35,609,414	*FAM117A*	Reproduction
10	78,274,732–78,283,255	*PER3*	Melatonin and core body temperature rhythms resynchronize
15	60,734,22–62,083,71	*HOXC10*	Regulate cell differentiation
15	62,437,71–62,716,81	*HOXC11*	Tail fat development
15	63,382,71–63,384,20	*HOXC12*	Tail fat development
15	63,426,27–64,605,57	*HOXC13*	Tail fat development
15	28,607,005–28,679,716	*TBK1*	Innate immune response
15	28,718,530–28,746,137	*ZC3H10*	Tail fat development
15	28,757,496–287,993,90	*ERBB3*	Normal growth and development
15	54,582,673–54,583,137	*CDK2*	Cell cycle regulation, cell proliferation and apoptosis
17	22,645,442–22,820,539	*MMP19*	Proliferation
17	29,540,625–29,557,094	*SNORA53*	Fat tailed development
17	29,631,331–29,661,343	*APAF1*	Immunity
17	34,461,385–34,517,413	*DDX47*	Wool Production
17	34,971,013–34,971,123	*LRP6*	Disease resistance
17	53,661,638–53,752,199	*PHB2*	Immunoprecipitation and embryo developments
17	53,803,322–53,859,050	*oar-mir-200c*	Milk production
17	53,875,546–53,895,506	*PTPN6*	Metabolism
2	58,319,311–58,656,152	*LGALS2*	Disease resistance
2	10,409,016,7–10,410,456,3	*H1-0*	Antibodies
2	10,481,607,5–10,500,749,7	*GCAT*	Regulate cardiac development
2	17,658,885,2–17,728,788,6	*SOX10*	Coat color patterns
2	18,434,313,9–18,434,327,5	*CRPPA*	Red meat production
2	19,005,983,1–19,006,799,7	*RELN*	Fat tailed deposition

**Table 5:** The candidate genomic regions and genes identified to be under selection by the XP-EHH approach for the comparison between the short fat-tailed (Yabuyi sheep) and log fat-tailed (Karakul sheep) breeds.

OAR	Gene Position (bp)	Gene Name	Function
**10**	35,583,842–35,609,414	*FGF9*	Early gonadal development/testosterone biosynthesis
**10**	35,786,789–35,798,732	*SKA3*	Disease resistance
**10**	35,862,425–35,885,746	*LATS2*	Pubertal development of male reproductive tract and spermatogenesis
**10**	36,045,326–36,103,818	*IFT88*	Reproduction
**11**	15,474,422–15,476,403	*CCL11*	Disease resistance
**11**	36,435,040–36,455,430	*FAM117A*	Regulation of ovulation rate
**11**	37,337,231–37,338,988	*HOXB13*	Tail formation
**11**	40,552,263–40,557,934	*KRT27*	Wool follicle and fiber morphology
**11**	46,503,733–46,5282,30	*MRC2*	Immunity
**14**	14,231,363–14,232,541	*MC1R*	Black coat color and pigmentation
**2**	72,847,41–75,096,31	*PAPPA*	Reproduction (follicle development)
**2**	12,325,968–12,488,187	*LPAR1*	Reproduction (embryo development)
**2**	14,235,503,1–14,244,934,8	*SCN1A*	Disease resistance (epilepsy)
**2**	24,675,580,7–24,686,431,8	*PAX7*	Regulation of myogenesis/proliferation
**24**	34,631,867–349,509,62	*CUX1*	Reproduction (Fetal development)
**3**	94,005,022–94,024,647	*CYP26B1*	Regulation of spermatogenesis
**3**	19,093,521,6–19,139,918,6	*SOX5*	Ear developmental processes
**3**	19,337,134,2–193,429,711	*GYS2*	Hepatocyte growth factor
**3**	19,343,685,7–19,344,439,8	*SPX*	Antibody response
**3**	20,164,430,6–20,166,437,1	*GSG1*	Fetal development
**3**	20,729,131,1–20,733,999,1	*C1R*	Immunity
**3**	20,735,340,8–20,736,655,7	*C1S*	Immunity
**3**	20,744,522,8–20,744,919,3	*PHB2*	Embryo development
**3**	20,745,499,6–20,746,386,0	*PTPN6*	Metabolism
**3**	20,758,319,4–20,760,861,0	*CD4*	Immunity (enhances antibody responses)
**3**	20,792,799,7–20,793,488,5	*LTBR*	Immune response
**3**	20,798,068,7–20,798,484,4	*TNFRSF1A*	Disease resistance
**3**	20,806,906,2–20,808,248,0	*CD9*	Various cellular processes (immune, growth, reproduction).
**3**	20,816,481,5–20,828,841,8	*VWF*	Disease resistance
**3**	20,830,234,2–20,864,109,0	*ANO2*	Antibody
**9**	13,460,501–13,464,151	*VPS28*	Growth factor
**9**	14,648,230–14,719,519	*ADGRB1*	Resistance to diarrhea
**9**	15,738,938–15,903,858	*PTK2*	Reproduction

## Supplementary Materials

The following supplementary materials are available online at https://www.mdpi.com/2073-4425/11/5/494/s1. Figure S1. Population structuring among the five Chinese local sheep breeds analyzed by the ADMIXTURE program. Figure S2. Linkage disequilibrium (LD) decay by genomic distances between SNPs in the five Chinese local sheep breeds. Figure S3. Maximum-likelihood phylogenetic tree of the five Chinese local sheep breeds reconstructed using the RAxML software with the GTRGAMMA model followed by a correction for ascertainment bias. Figure S4. A neighbor-joining phylogenetic tree of the five Chinese local sheep breeds reconstructed using the MEGA7 package. Table S1. Description of the five Chinese local sheep breeds and their agro-ecological locations. Table S2. Pairwise population differentiation (F_ST_) between the five Chinese local sheep breeds. Table S3. The top 1% candidate genes detected by the Z(F_ST)_ approach. Table S4. GO terms and KEGG pathways of the top 1% Z(F_ST_) values. Table S5. Candidate genes detected by the top 1% XP-EHH values. Table S6. GO terms and KEGG pathways of the top 1% XP-EHH values. Table S7. GO terms identified based on the F_ST_ approach between the short fat-tailed (Yabuyi sheep) and long fat-tailed (Karakul sheep) breeds. Table S8. GO terms identified based on the XP-EHH approach between the short fat-tailed (Yabuyi sheep) and long fat-tailed (Karakul sheep) breeds.

## Data Accessibility

The datasets analyzed on this study are available upon request from corresponding authors.
