# Supplementary material for: Genomic Diversity, Population Structure, and Signature of Selection in Five Chinese Native Sheep Breeds Adapted to Extreme Environments
Source: Genes (Basel). 2020 Apr 30;11(5):494. doi: 10.3390/genes11050494 (PMC7290715; doi:10.3390/genes11050494)
Supplement: Supplementary file 1 [file genes-11-00494-s001.zip › Figure S2.docx]

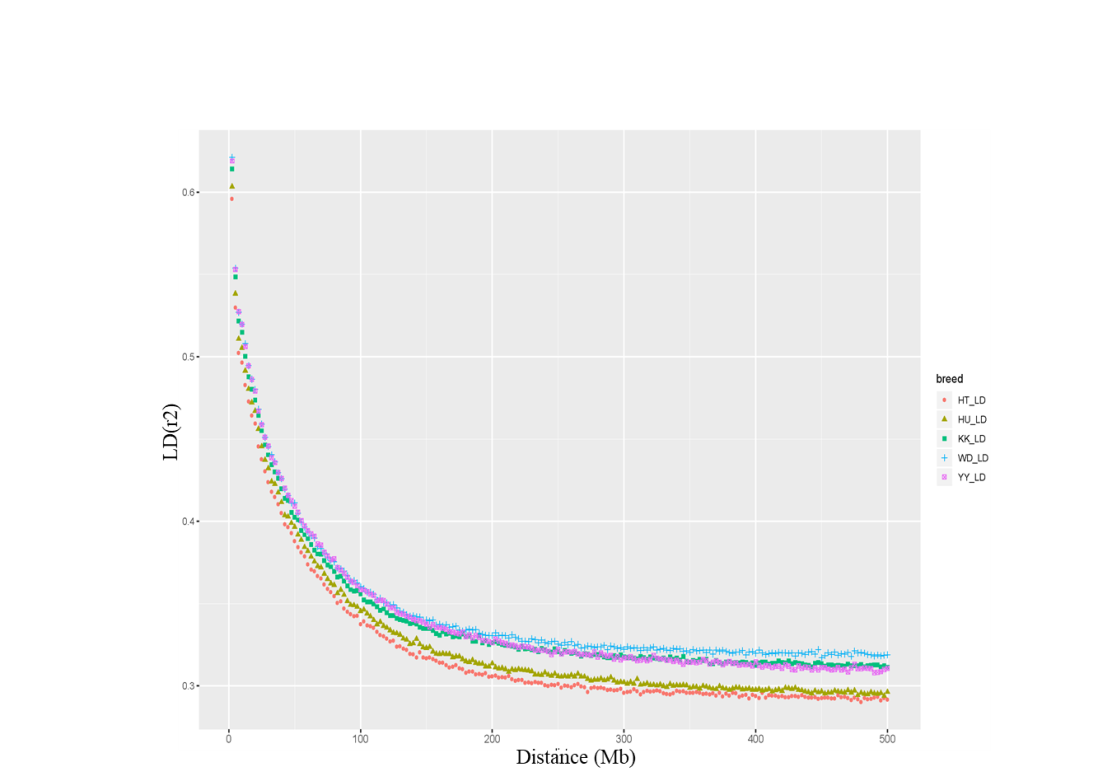


Figure S2. Linkage disequilibrium (LD) decay by genomic distances of SNPs in the five Chinese local sheep breeds. Hetian (HT), Hu (HU), Karakul (KK), Wadi (WD), and Yabuyi (YY).
