# Supplementary material for: Genomic Diversity, Population Structure, and Signature of Selection in Five Chinese Native Sheep Breeds Adapted to Extreme Environments
Source: Genes (Basel). 2020 Apr 30;11(5):494. doi: 10.3390/genes11050494 (PMC7290715; doi:10.3390/genes11050494)
Supplement: Supplementary file 1 [file genes-11-00494-s001.zip › Figure S3.docx]

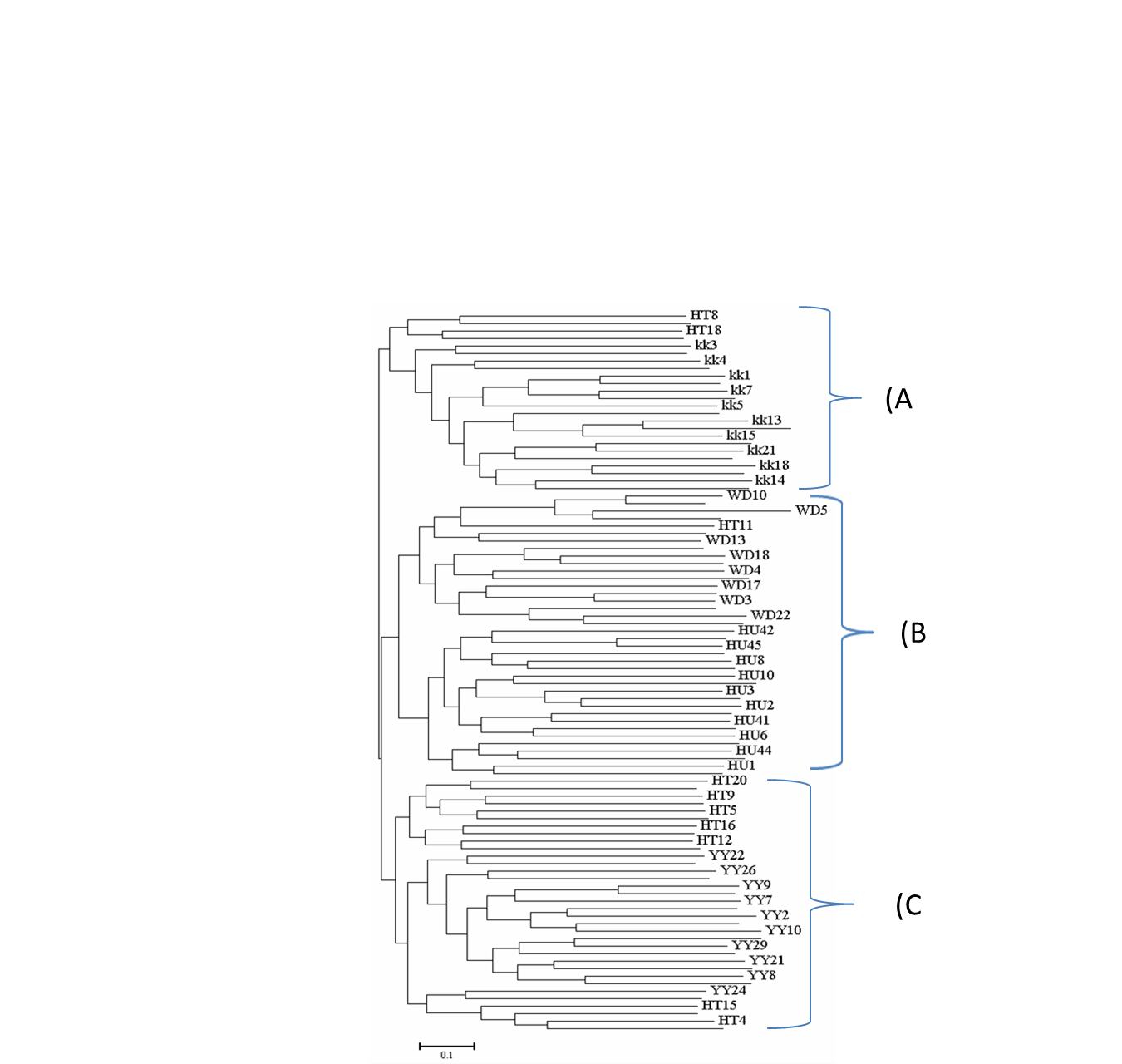


Figure S3. A) Maximum-likelihood phylogenetic tree of the five Chinese local sheep breeds reconstructed using the RAxML software with the GTRGAMMA model followed by a correction for ascertainment bias. In the phylogenetic tree, the first or Clade A of sheep group contained Karakul breed and some individuals of Hetain breed; the second or Clade B composed of Hu and Wadi breeds, which appeared as sister taxa to each other; and the third or Clade C of sheep grouped Yabuyi breed with some individuals of Hetian breed.


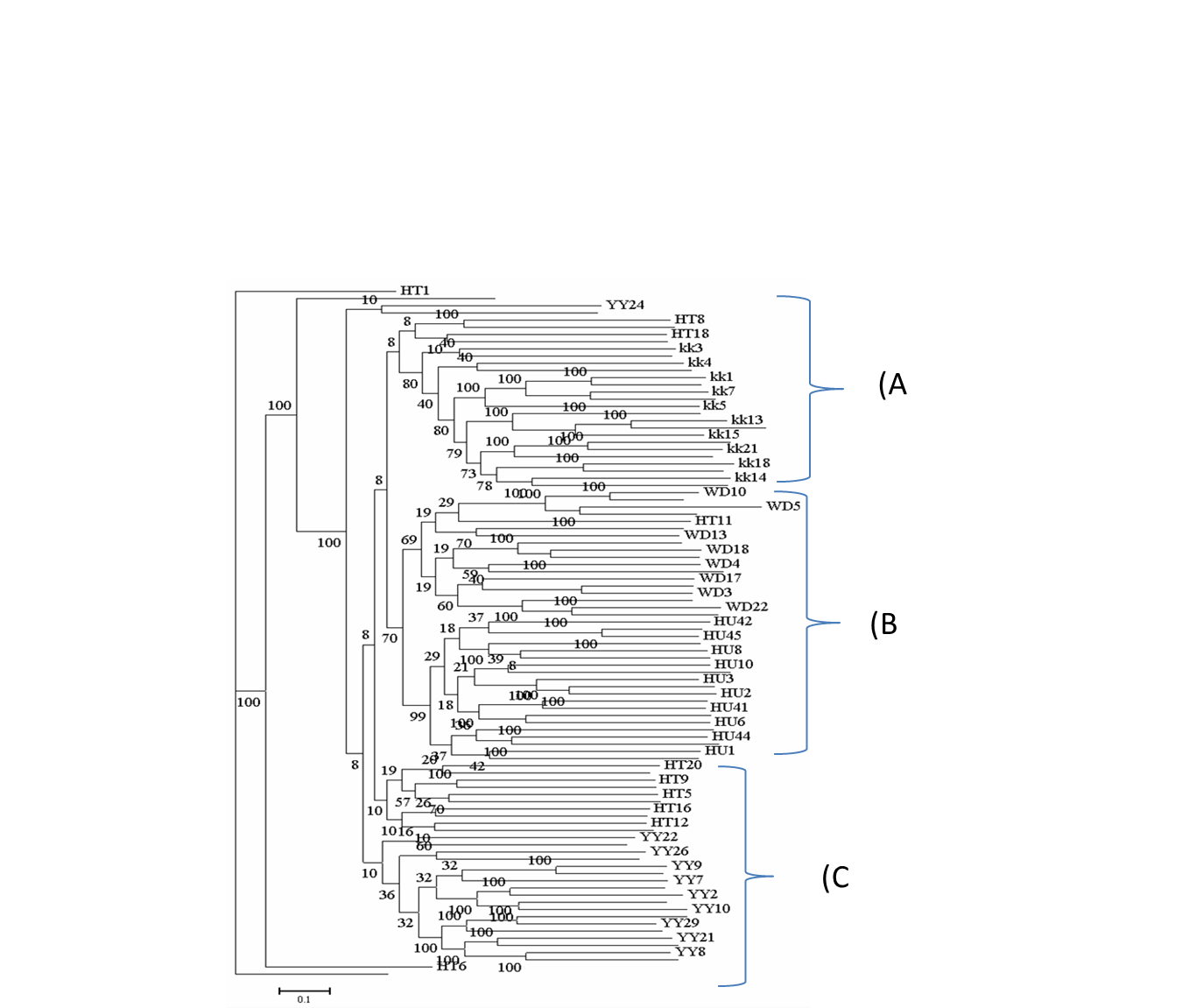


Figure S3. B) Maximum-likelihood phylogenetic tree of the five Chinese local sheep breeds reconstructed using the RAxML software with the GTRGAMMA model followed by a correction for ascertainment bias. In addition to above descriptions of Figure S3A, some of the clusters in the ML tree have low values of bootstraps and thus they are not fully statistically supported.
