# Supplementary material for: Genomic Diversity, Population Structure, and Signature of Selection in Five Chinese Native Sheep Breeds Adapted to Extreme Environments
Source: Genes (Basel). 2020 Apr 30;11(5):494. doi: 10.3390/genes11050494 (PMC7290715; doi:10.3390/genes11050494)
Supplement: Supplementary file 1 [file genes-11-00494-s001.zip › Figure S4.docx]

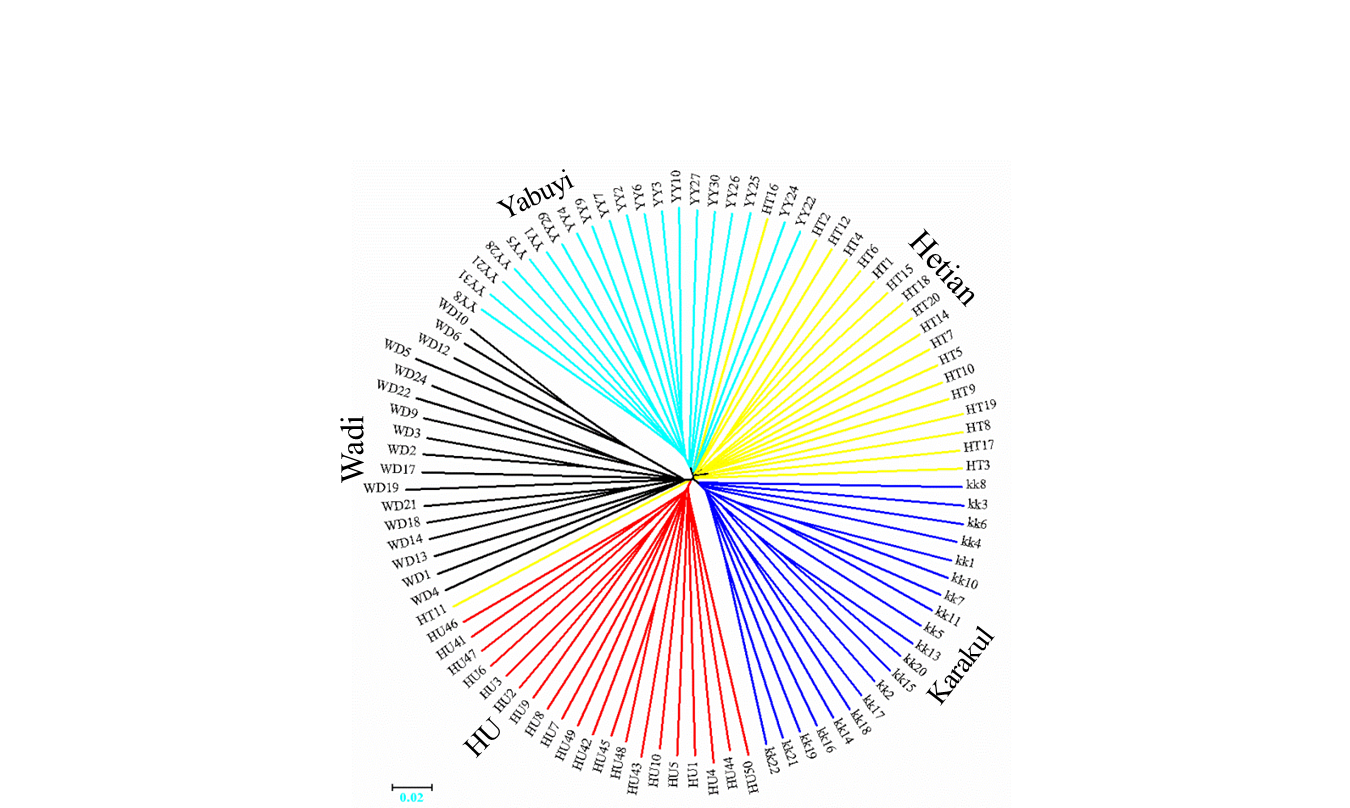


**Figure S4**. A neighbor‐joining phylogenetic tree of five native Chinese sheep breeds reconstructed using the MEGA7 package. The sheep breeds clustered into three groups based primarily on their tail-types and geographical distributions.
