# Supplementary material for: Genomic Diversity, Population Structure, and Signature of Selection in Five Chinese Native Sheep Breeds Adapted to Extreme Environments
Source: Genes (Basel). 2020 Apr 30;11(5):494. doi: 10.3390/genes11050494 (PMC7290715; doi:10.3390/genes11050494)
Supplement: Supplementary file 1 [file genes-11-00494-s001.zip › Table S1.docx]

**Table S1**. Description of five Chinese local sheep breeds and their agro-ecological locations.

| **Breed** | **Hetian (HT)** | **Karakul (KK)** | **Yabuyi (YY)** | **Wadi (WD)** | **Hu (HU)** |
| --- | --- | --- | --- | --- | --- |
| Coat colour | White with pigmentation | Mainly black or grey | White | Mostly white with pigmentation | White with pigmentation |
| Body size | Small | Medium | Small | Big | Large |
| Body length | Long | Stand tall | Long | Medium | Long |
| Hair/wool length | Long wool | Long wool | Medium | Short wool | Variable wool length |
| Tail type | Short fat-tail | Long fat-tail | short fat-tail | Short fat-tail | Short fat-tail |
| Use | Meat/carpet wool | Lamb skin/meat | Meat | Meat/wool | Meat/white lamb skin |
| Agro-ecology | Arid, low-land | Arid, semi-desert, low-land | Arid, low land | Sub-humid, low-land | Moist, low-land |
| Geographic location | Xinjiang/Hetian | Xinjiang | Xinjiang | Shandong/Binzhou | Jiangsu /Xuzhou |
| Temperature (^O^C) | -28.9~43.2 | -20~33 | -26~43 | -16~39 | -9.9~38.3 |
| Average annual rain fall (mm) | 150 | 150 | 35 | 592 | 653.3 |
