# Supplementary material for: Genomic Diversity, Population Structure, and Signature of Selection in Five Chinese Native Sheep Breeds Adapted to Extreme Environments
Source: Genes (Basel). 2020 Apr 30;11(5):494. doi: 10.3390/genes11050494 (PMC7290715; doi:10.3390/genes11050494)
Supplement: Supplementary file 1 [file genes-11-00494-s001.zip › Table S2.docx]

**Table S2**. Pairwise population differentiation (F_ST_) between the five Chinese local sheep breeds.

| Breed | Hetian | Hu | Karakul | Wadi | Yabuyi |
| --- | --- | --- | --- | --- | --- |
| Hetian | - |  |  |  |  |
| Hu | 0.025 | - |  |  |  |
| Karakul | 0.031 | 0.047 | - |  |  |
| Wadi | 0.025 | 0.035 | 0.048 | - |  |
| Yabuyi | 0.022 | 0.045 | 0.054 | 0.045 | - |
